# Application of Statistical Methods for Central Statistical Monitoring and Implementations on the German Multiple Sclerosis Registry

**DOI:** 10.1007/s43441-023-00550-0

**Published:** 2023-07-14

**Authors:** Firas Fneish, David Ellenberger, Niklas Frahm, Alexander Stahmann, Gerhard Fortwengel, Frank Schaarschmidt

**Affiliations:** 1 Department of Biostatistics, Institute of Cell Biology and Biophysics, Leibniz University Hannover, Herrenhäuser Straße 2, 30419 Hannover, Germany; 2 German MS-Register, MS Forschungs- und Projektentwicklungs- gGmbH [MSFP], Krausenstraße 50, 30171 Hannover, Germany; 3 Faculty III–Media, Information, and Design, Hochschule Hannover, 30539 Hannover, Germany

**Keywords:** Monitoring, Data quality control, Multicenter clinical trials, Grand mean, Registry data

## Abstract

Monitoring of clinical trials is a fundamental process required by regulatory agencies. It assures the compliance of a center to the required regulations and the trial protocol. Traditionally, monitoring teams relied on extensive on-site visits and source data verification. However, this is costly, and the outcome is limited. Thus, *central statistical monitoring* (CSM) is an additional approach recently embraced by the International Council for Harmonisation (ICH) to detect problematic or erroneous data by using visualizations and statistical control measures. Existing implementations have been primarily focused on detecting inlier and outlier data. Other approaches include principal component analysis and distribution of the data. Here we focus on the utilization of comparisons of centers to the Grand mean for different model types and assumptions for common data types, such as binomial, ordinal, and continuous response variables. We implement the usage of multiple comparisons of single centers to the Grand mean of all centers. This approach is also available for various non-normal data types that are abundant in clinical trials. Further, using confidence intervals, an assessment of equivalence to the Grand mean can be applied. In a Monte Carlo simulation study, the applied statistical approaches have been investigated for their ability to control type I error and the assessment of their respective power for balanced and unbalanced designs which are common in registry data and clinical trials. Data from the German Multiple Sclerosis Registry (GMSR) including proportions of missing data, adverse events and disease severity scores were used to verify the results on Real-World-Data (RWD).

**Supplementary Information:**

The online version contains supplementary material available at 10.1007/s43441-023-00550-0.

## Introduction

Multicenter clinical trials are imperative to obtain a conclusive assessment concerning the safety and efficacy of medical treatments. They involve diverse clinics or hospitals, and their respective personnel [1]. This requires the monitoring team to ensure the compliance of each center to the study protocol and the requirements of good clinical practice. Compliance of a center to the required regulations will make the center’s data more reliable. Non-compliance events may lead to errors in patient inclusion criteria, operating procedures and to various types of data entry errors [2, 3]. Additionally, data tampering or fraud may occur in a single center [4]. All these difficulties may result in biased estimates of the investigated treatment efficacy as well as to false positive or false negative detection of safety issues. The monitoring team traditionally performs on-site visits to each study center to ensure compliance of the regulatory requirements; however, these activities have been reported to be costly and of limited outcome with regards to data quality [5, 6]. In the preceding years, central statistical monitoring (CSM) was proposed as an amendment to a thorough source data verification (SDV) that requires on-site visits [7, 8].

CSM utilizes graphical approaches, summary statistics and statistical tests to assess incoming data from all centers in the trial [9–11]. The assessment of center compliance can be achieved by statistical models to assess adherence levels. The primary aim is to detect data entry errors, adverse event rates in single centers or safety issues related to individual patients. Moreover, CSM serves to identify centers that could require additional monitoring activities due to deviations or outlier detection. A robust risk assessment of the key risk indicators (KRIs) in clinical trials can target onsite-monitoring activities [12, 13]. Risk assessment prior to trial initiation can facilitate whether an onsite-monitoring technique or CSM technique is needed to monitor a certain risk. Timmerman et al. (2016) illustrates how CSM can be a means to identify KRIs to target adaptive monitoring [14].

Numerous statistical methods have been applied for monitoring approaches for the implementation of CSM [9, 15, 16]. Based on covariate type, statistical methods were applied to detect atypical/outlier data. For the purpose of risk based centralized monitoring, classical statistical methods have been categorized as unsupervised and supervised monitoring techniques [17]. Existing publications on CSM focused on outlier/inlier detection on different levels e.g., center, country and regional and demonstrated the usage of principle component analysis on the center level.

However, single centers in multicenter clinical trials might deviate from the study protocol or inclusion criteria. They might also deviate in clinical practice, or there might be misunderstandings concerning the definition of adverse events or categorical variables or disease severity scores to be recorded. Such deviations will not produce single extreme values in the data. They will rather lead to deviating summary statistics, adverse event rates, or class frequencies of categorical data. Desmet et al. (2014) proposed the usage of linear mixed effects models to detect location differences between center and other centers for a continuous outcome and a beta binomial model for proportion comparison for a certain event in a center [18, 19]. In the following paper we propose to use multiple comparisons of single centers to the Grand mean (GM) of all centers. This approach is available for various data types that are abundant in clinical trials. It can be used to detect centers that are significantly deviating from average. Further, confidence intervals are available, such that an assessment of equivalence to the average can be applied. Center comparisons to the GM of the data has been an overlooked aspect. In the following, we will firstly define comparisons to the GM for different model types and assumptions for common data types, such as binomial, ordinal, and continuous response variables. Generalized linear models (GLM), bayesian generalized linear models (BayesGLM), and bias-reduced generalized linear models (BrGLM) were applied for binomial outcomes. For continuous outcomes, a non-parametric and a linear approach are investigated. As for ordinal data, a non-parametric approach is assessed. The correction for multiple testing is accounted for when performing the contrasts. Since approaches are asymptotic and thus depend on the sample size, they were investigated in a Monte Carlo simulation for their ability to control the type I error ($\alpha$) and achieve the highest possible power ($1-\beta$). We demonstrate the implementation of these methods on examples based on data from the German Multiple Sclerosis Registry (GMSR) [20].

## Real-World Data from GMSR

CSM aids clinical trials and registries in data monitoring for many variables. GMSR collects data directly from participating centers through a certified web-based data capture (EDC) system. The data collected includes a wide range of variables such as patient profile, disease status and medication treatments. We refer to Ohle et al. (2021) for further details on the GMSR [20]. We included centers that are participating in the pharmacovigilance module at the GMSR each having at least 50 patients under observation in the database. An overview of the GMSR data is shown in Table 1 for specific variable types considered in this research.

**Table 1:** Basic and Clinical Characterization of Patients Part of Pharmacovigilance Module in the GMSR Data at the Latest Visit

Age of onset (median, quantiles)	30.25 (23.42, 38.42)
Missing age of onset	153 (8.1%)
Sex	
Females	1356 (72%)
Males	538 (28%)
Adverse events reported	
Number of adverse events reported	232
Number of patients experiencing adverse evenets	183
Disease course (at latest visit)	
RRMS	1623 (85.7%)
SPMS	237 (12.5%)
CIS	15 (0.8%)
Unknown	19 (1)

**Figure 1:**
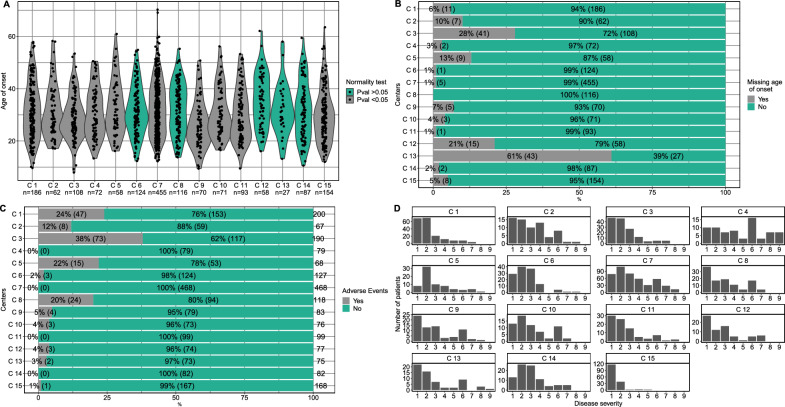
GMSR Data Stratified by Center for Four Variables **A**–**D**. **A** Violin plot including kernel density estimates of age at disease onset indicating possible violations of normality. **B** Missing age of onset (%) in patients without queries. **C** Reported number of patients having adverse events (%). **D** Histograms of disease severity (EDSS) for patients at latest visit.

Figure 1 shows the dataset of three variables for each center. The dataset covers age at onset, adverse events (AE), expanded disability status scale (EDSS) representing continuous, binomial, and ordinal data types respectively. EDSS and AE are reported for each visit. Figure 1a shows the distribution of patients’ age at onset and highlights that data may not be normally distributed. Shapiro test was used to indicate whether the data of individual centers follow the normal distribution. Violations of the normality assumption suggest the need for non-parametric methods to perform center comparisons to the GM. For the same variable Fig. 1b shows the missingness found in each center. It illustrates the center performance in terms of data completeness. Although it is common to have missing data, the question arises at what level it is unacceptable? Similarly for adverse events, one center (C3) reports 38% of patients having adverse events while other centers range between 0 and 24% (Fig. 1c), this observation again designates a variation in the proportions for a certain event between centers and shows the need for a test to hint for the problematic center(s). As for EDSS measurements (Fig. 1d) it exhibits a clear difference for disease severity for patients between centers.

The visualization of these variables provides to the stakeholders an overview of the data at hand. However, it does not directly pinpoint or highlight a problematic center. Although the observed differences between centers could be natural due to patient variation or other factors, it is essential to confirm deviating centers at a given statistical certainty. In some cases, inference of a center being problematic can only be deduced with appropriate statistical testing e.g., complex multicenter clinical trial. This dataset will be used to demonstrate comparisons of the individual mean center to the GM of all centers for different scales of measurement.

## Materials and Methods

We consider a wide spectrum of scenarios relevant to registry and clinical trial data with several centers for the response outcome variable. Let $i$ be the index of the centers in a clinical trial$i=1,\dots , I$. Within each center $i$ there are ${n}_{i}$ subjects, with subject index$j=1,\dots ,{n}_{i}$. The GM of all centers within the trial is denoted by ${\widehat{m}}_{.}$.

### Comparisons to ${\widehat{m}}_{.}$

For a given model with parameters *m*_*i*_ and possibly unbalanced samples sizes ${n}_{i}$ the GM ${m}_{.}$ can be computed by ${m}_{.}=\sum_{i=1}^{I}\frac{{n}_{i}}{N}{m}_{i}$, where* N* is the total sample size, $N=\sum_{i=1}^{I}{n}_{i}$. Comparisons of each centers parameter *m*_*i*_ to the GM *m*_*.*_ can then be written as a set of *k* = *1,…,K* linear contrasts, with contrast coefficients ${c}_{k}=\left({c}_{k1}, {c}_{k2},{c}_{k3},\dots ,{c}_{kI}\right)$:

$$\begin{gathered} c_{1} = \left( {1 - \frac{{n_{1} }}{N}, - \frac{{n_{2} }}{N}, - \frac{{n_{3} }}{N}, \ldots , - \frac{{n_{I} }}{N}} \right) \hfill \\ c_{2} = \left( { - \frac{{n_{1} }}{N}, 1 - \frac{{n_{2} }}{N}, - \frac{{n_{3} }}{N}, \ldots , - \frac{{n_{I} }}{N}} \right) \hfill \\ \ldots \hfill \\ c_{K} = \left( { - \frac{{n_{1} }}{N}, - \frac{{n_{2} }}{N}, - \frac{{n_{3} }}{N}, \ldots , 1 - \frac{{n_{I} }}{N}} \right) \hfill \\ \end{gathered}$$

The deviation of the *k*th center from the GM can then be written as:

$${d}_{k}={m}_{i=k}-{m}_{.}=\sum_{i=1}^{I}{c}_{ki}{m}_{i}$$

Written in this way, the comparisons to GM are a special case of the framework of testing general linear hypotheses [21]. In this framework it is possible to perform hypotheses tests adjusted for multiple comparisons and to compute simultaneous confidence intervals for the parameters defined by the contrasts.

In this application, it can be of interest to test the null hypothesis that no center deviates from the overall mean,

$${H}_{0}: {d}_{k}={(m}_{i=k}-{m}_{.})=0,\mathrm{\ for\ all\ }k=1,\dots K,$$

versus the alternative hypothesis that at least one center deviates from the overall mean,

$${H}_{A}: {d}_{k}={(m}_{i=k}-{m}_{.})\ne 0,\mathrm{\ for\ at\ least\ one\ }k=1,\dots K.$$

In some cases, a test decision concerning a significant deviation might not be of interest. Like in tests on equivalence, the objective can be to infer whether single centers do not show a relevant difference from the overall mean. In this case, a prior definition of relevant deviations or equivalence margins,$\left[-\delta , \delta \right]$, has to be specified based on subject knowledge. Then, it can be inferred whether the upper and lower confidence limits for each center’s deviation *d*_*k*_ are included in this range or not.

For the full details of computing p-values of the above hypothesis tests and simultaneous confidence intervals, we refer to Hothorn et al. (2008) [21]. The most important steps from Hothorn et al. (2008) are outlined below. Stacking the *k* = *1,…,K* vectors of contrast coefficients, *c*_*k*_, yields a contrast matrix *C* with *K* rows and *I* columns. Fitting linear or generalized linear models yields a vector of estimates of the model parameters with elements ${\widehat{m}}_{i}$, ${\widehat{m}=\left({\widehat{m}}_{1},{\widehat{m}}_{2},{\widehat{m}}_{3},\dots ,{\widehat{m}}_{I}\right)}^{T}$ and the corresponding estimated variance–covariance matrix of model parameters, $\widehat{V}$. Estimates for the deviations of centers from the GM are then $\widehat{d}=C\widehat{m}$, the corresponding variance–covariance matrix of these deviations is $\widehat{U}=C\widehat{V}{C}^{T}$, where ^*T*^ denotes a transposed vector or matrix. The estimated variance of the elements ${\widehat{d}}_{k}$ in $\widehat{d}=\left({\widehat{d}}_{1},{\widehat{d}}_{2},{\widehat{d}}_{3}\dots , {\widehat{d}}_{K}\right)$ are the diagonal elements of $\widehat{U}$, $\widehat{u}=diag(\widehat{U})$, with elements ${\widehat{u}}_{k}$. Their square roots are then the estimated standard errors of the ${\widehat{d}}_{k}$, that is, $\widehat{se}\left({\widehat{d}}_{k}\right)=\sqrt{{\widehat{u}}_{k}}$. Finally, the estimated correlation matrix $\widehat{R}$ of $\left({\widehat{d}}_{1},{\widehat{d}}_{2},{\widehat{d}}_{3}\dots , {\widehat{d}}_{K}\right)$ follows from standardizing the matrix $\widehat{U}$ with its diagonal elements $\sqrt{{\widehat{u}}_{k}}$.

Tests of the hypotheses presented above are then based on the test statistics ${t}_{k}=\frac{{\widehat{d}}_{k}}{\widehat{se}\left({\widehat{d}}_{k}\right)}$, the corresponding adjusted p-values are computed from a multivariate *t*-distribution (or asymptotically from a multivariate normal distribution) with correlation matrix $\widehat{R}$, for linear models or generalized linear models, respectively.

Simultaneous confidence intervals for each center’s deviation from GM, ${\widehat{d}}_{k}$, can be computed using the formula

$$\left[{\widehat{d}}_{k}\pm {q}_{1-\alpha ,two-sided, \widehat{R}}\widehat{se}\left({\widehat{d}}_{k}\right)\right]=\left[{(\widehat{m}}_{i=k}-{\widehat{m}}_{.})\pm {q}_{1-\alpha ,two-sided, \widehat{R}}\widehat{se}\left({\widehat{m}}_{i=k}-{\widehat{m}}_{.}\right)\right]$$

where ${q}_{1-\alpha ,two-sided, \widehat{R}}$ is the two-sided equicoordinate (1 − $\alpha$) quantile of multivariate *t* or multivariate normal distribution, respectively. For further details of computing adjusted p-values and quantiles of multivariate *t* and normal distributions, we refer to Genz and Bretz (2009) [22].

For a thorough data interpretation, merely relying on rejection/non-rejection at one significance at level, say 0.05, or merely relying on the presented *p*-values is discouraged (e.g. ASA statement on *p*-values, Wasserstein and Lazar 2016) [23]. Rather, estimated effects (here, deviations from Grand mean) and the corresponding confidence limits should be displayed and used for interpretation: then, the relevance of observed effects can be assessed, or, non-inferiority or equivalence can be assessed based on inclusion of confidence limits in pre-specified equivalence margins for the corresponding parameter.

### Response Variables

#### Continuous Outcomes

Continuous data may follow the normal distribution, possibly after a suitable data transformation to achieve normality and homogeneous variances. It can then be analyzed by the model used in one-way analysis of variance.

$${Y}_{ij} \sim {m}_{i}+{\varepsilon }_{ij} , {\varepsilon }_{ij} \sim N(0, {\sigma }^{2})$$

Here, ${m}_{i}$ is the expected value of center $i$. In this case, the above multiple comparison procedure is well established and exact. In case of continuous outcomes which are in contradiction to normality before and after transformations, a non-parametric method is described in section "Non-parametric Approach for Multiple Comparisons (Nparcomp) for Continuous and Ordinal Endpoints" as an alternative.

#### Binomial Outcome

The number of events ${Y}_{i}$ is assumed to follow a binomial distribution in which ${\pi }_{i}$ is the event probability in a center *i*.

$${Y}_{i}\sim Binomial({n}_{i},{\pi }_{i})$$

For binomial data, we will assume that a generalized linear model (GLM) is fitted with the canonical logit link:

$${m}_{i}= \mathrm{log}(\frac{{\pi }_{i}}{1-{\pi }_{i}})$$

Thus, comparisons to GM will be performed on the logit scale [24].

#### Excess 0 s in Binomial Data

Fitting a classical generalized linear model for binomial data with zero excess ${Y}_{i}=0$ successes/failures is a common problem in different scientific fields such as clinical trials and toxicological experiments [25]. As soon as ${Y}_{i}=0$ in one or several centers, numerically ${m}_{i}=\mathrm{log}(\frac{{\pi }_{i}}{1-{\pi }_{i}})$ becomes very small and $se({m}_{i})$ will be very large. Several alternatives are available to avoid extreme $se$. In the next subsection we consider two alternatives, a Bayesian linear model [26] and the Bias-reduced generalized linear model[27].

### Estimators and Models Assumptions

#### Bayesian Generalized Linear Models (BayesGLM) for Binomial Endpoint

The first approach we consider for dealing with zero excess binomial data ${Y}_{i}=0$ in one or several centers is a Bayesian linear model with non-informative priors. Gelman et al. (2008) used scaled Cauchy distributions as priors for each model parameters that estimate effects, e.g. differences on the logit scale. Cauchy priors for a model parameter entail the assumption that extreme center effects on the logit scale are implausible. Prior assumptions for a baseline risk or control group allows a wider range such that ${10}^{-9}$ < $\mathrm{\pi }_{i}$ < ${1-10}^{9}$ (Gelman et al. 2008) [28]. Prior assumptions on parameters impose a restriction on the parameter estimation; this prevents that estimated parameter from becoming extreme and thus prevents the standard error from becoming extreme as well.

#### Bias-Reduced Generalized Linear Models (BrGLM) for Binomial Endpoint

A second option to account for binomial data with 0 excess observations ${Y}_{i}=0$ in one or several centers is a bias reduced GLM [26]. In this approach, the iteratively reweighted least square algorithm used for fitting generalized linear models is modified by adding pseudo-observations depending on the estimated parameters, such that bias is reduced iteratively [29]. This approach always leads to finite estimates of the logits *m*_*i*_, and of its related variance covariance matrix $\widehat{V}$, such that computation can proceed as described in earlier sections. For the computational details we refer to Kosmidis and Firth, 2009 [27], and Kosmidis and Firth (2021) [29].

#### Non-parametric Approach for Multiple Comparisons (Nparcomp) for Continuous and Ordinal Endpoints

Konietschke et al. (2012) proposed a non-parametric procedure to perform general multiple contrast tests between several samples without relying on assuming any specific distribution for the data ${Y}_{ij}$ [30]. Very briefly, they assume that the data are independent realizations ${Y}_{ij}\sim {F}_{i}$, where the ${F}_{i}$ denote, in our context, the distributions in centers $i=1,\dots , I$. These distribution functions need not to be explicitly specified, they may differ between centers, including cases like heteroscedastic data, or different levels of skewedness between centers. Their procedure further allows ${Y}_{ij}$ to be heavily tied data, including ordinal data, such as disease severity scores. The comparisons between centers rely on the generalized relative effects ${\pi }_{i}$, which are defined as the probability that observations from center $i$ is lower or equal than an observation from the average distribution $G$ resulting from the averaging ${F}_{i}$ across all centers. Applying the above contrast matrix C allows to compute adjusted p-values for the deviations of centers from the average as well as simultaneous confidence intervals, again using multivariate-*t*- (or -normal-) distribution for the test statistics derived from the generalized relative effects. For full computational details we refer to Konietschke et al. (2012).

The method of Konietschke et al. (2012) is an asymptotic one, in other words the control of type I error for small samples is unclear. Specifically, Konietschke et al. (2012) state that convergence to normality is slow, especially for many groups (i.e. centers) and small sample sizes. Their simulation study only includes cases with $i=3, 4, 5$ groups, and only mildly unbalanced sample sizes. Moreover, their simulation study involved only continuous data, while results for highly discrete ordinal data were not shown. In application to real data with ordinal variables, we observed simultaneous confidence intervals indicating quite clear deviations from the null hypotheses, when sample sizes were extremely small. We therefore ran additional simulation studies specifically tailored for the applications described in this paper.

### Simulation Study

A Monte Carlo simulation study was performed to assess the control of type I error ($\alpha$), the probability to reject ${H}_{0}$ for at least one center if no center deviates from GM in which ${H}_{0}:$ ${m}_{i}-{m}_{.}=0$, and the power ($1-\beta$) against an alternative hypothesis ${H}_{A}:$ ${m}_{i}-{m}_{.}\ne 0$ for each method applied on its respective data type, the power represents the probability to reject ${H}_{0}$ for at least one center if ${H}_{A}$ is true for at least one center. Both GLM and Nparcomp are valid asymptotic methods that require large sample sizes; however, we are interested in their performance under small and unbalanced sample sizes. Since GLM has computational problems when ${Y}_{i}=0$, it is additionally compared to the alternative approaches (BayesGLM and BrGLM) under same settings. Here, power comparisons are of special interest as the three approaches handle the case of ${Y}_{i}=0$ differently. Nparcomp is also assessed when applied to ordinal data with few categories and small sample sizes. Ordinal outcome was simulated from normally distributed data which was then round to 0 digits to create discrete ordinal data.

Simulations were run for balanced and unbalanced designs with varying parameter settings: *I* = (5, 10) for number of centers in a trial, subjects per center varied between balanced and unbalanced scenarios of ${n}_{ij}$ = (2, 3, 4, 5, 6, 10, 20, 40, 50, 80, 100, 150, 200, …, 4000). Complete list of parameter settings for all simulations are available in the supplementary material. The ${n}_{i}$ in power simulations for unbalanced designs, deviating center constantly had half the number of observations as in other individual centers for covered scenarios, some additional scenarios where run for extreme small ${n}_{i}$ in deviating center. For continuous and ordinal power simulations, the true difference between means $(\delta$) were chosen such that for a given sample size a power of 80% is achieved in a two-sample *t*-test thus one center had a $\delta$ deviating from other centers. As for binomial power simulations, the success proportions of centers were chosen such that for a given sample size, a power of 80% is achieved in a two-sample proportion test, consequently, centers had a different success proportion from deviating center. For each parameter setting, a number of 1000 datasets were generated and tested by each method. Note that, with 1000 simulation runs to estimate the type I error, the standard error of an estimated type I error is $\approx 0.007$ and 95% of simulation results are expected in the range $\left[0.036, 0.063\right]$ if a method accurately controls type I error at $\alpha =0.05$.

#### Software and Packages

All simulations were performed in R, version 4.0.5. Implemented methods Linear Model, Generalized Linear Model, Bayesian Generalized Linear Model, Bias Reduction in Binomial response Generalized Linear Models and Non-parametric multiple comparisons are available in R-packages stats v4.0.5 [31] (R-core Team), arm v1.11-1 [32], brglm v0.6.2 [33] and nparcomp v3.0 [34] respectively. To compute GM contrasts, “multcomp” package was used [35].

## Results

This section shows the results of the simulation study. We describe first the results of the type I error control simulations and then the results of power simulations in comparison contrast to the GM.

### Simulations of Type I Error

**Figure 2:**
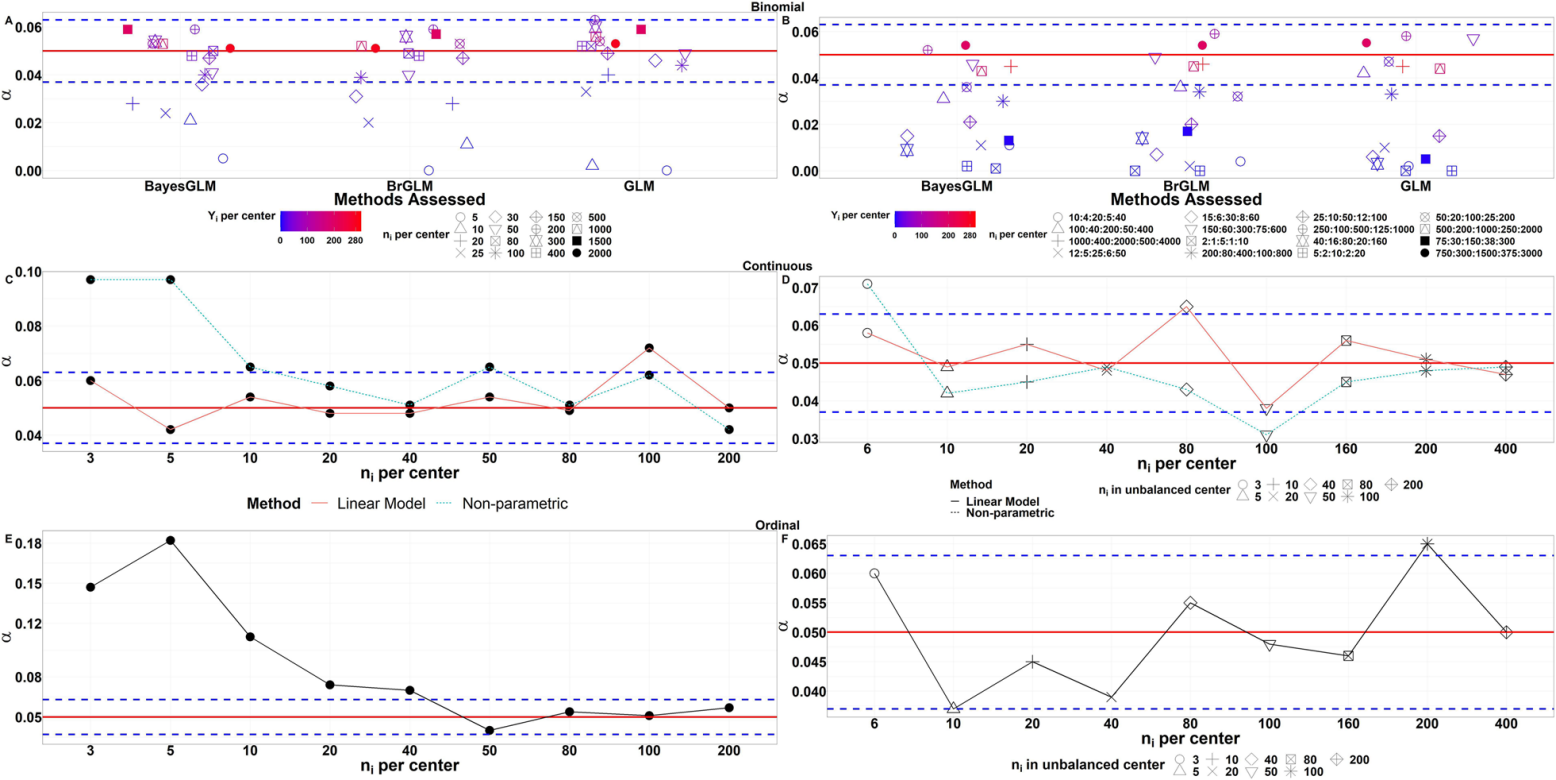
The Probability of Falsely Rejecting the Null Hypothesis for At Least One Center as a Function of Sample Size for Each Method Applied on Relevant Response Outcome for Balanced (Left Panel) and Unbalanced Designs (Right Panel). The nominal type I error rate ($\alpha =0.05$) is shown as a horizontal line. Dotted blue lines indicate error margins for simulations with 1000 runs. Simulated type I errors falling outside [0.037; 0.063] indicate a significant deviation from the prespecified level alpha = 0.05). BayesGLM Bayesian Generalized Linear Model, BrGLM Bias-reduced Generalized Linear Model, GLM Generalized Linear Model.

The simulations of type I error for all methods are shown in Fig. 2. For a binomial outcome, Fig. 2a shows the experimental setup used for a balanced design. Simulations show as the sample size per center N increases with increasing success probability of a certain response variable, the more a 5% rejection rate is achieved. For events with a low expected number of events $({n}_{i}{\pi }_{i})$ all three methods tend to show $\alpha$ below the nominal level. While for unbalanced designs, Fig. 2b shows no difference between the three methods. In extreme settings however, i.e. centers having a smaller number of patients compared to other centers that have a smaller success probability of a certain response variable, the methods appear to be conservative in achieving a 5% rate. For continuous outcomes, Fig. 2c and d show the simulations of linear model as a comparator to the non-parametric approach for balanced and unbalanced experimental design respectively. As anticipated, the non-parametric method shows increased type I error for small sample sizes [3, 5, 10]. A linear model is known to control the familywise type I error rate, the purpose of this comparison is to show the ability of the non-parametric method to control the type I error similarly to the linear model, specifically for extreme settings. For extreme settings such as centers with < 10 patients per center, the non-parametric method rejects up to 10% for balanced designs, however its control is sounder for unbalanced designs for all covered scenarios. For ordinal outcome, Fig. 2e and f show the simulations of the non-parametric method for balanced and unbalanced experimental designs respectively. Similarly, to the continuous outcome, for centers having < 10 patients the control of type error reaches to 18% for balanced designs and is maintained for all scenarios of unbalanced designs. Additional simulations with 5000 runs were performed for the non-parametric method with the same settings, similar type I error control is observed to the 1000 runs (Supplementary Fig. 1).

### Power Simulations

**Figure 3:**
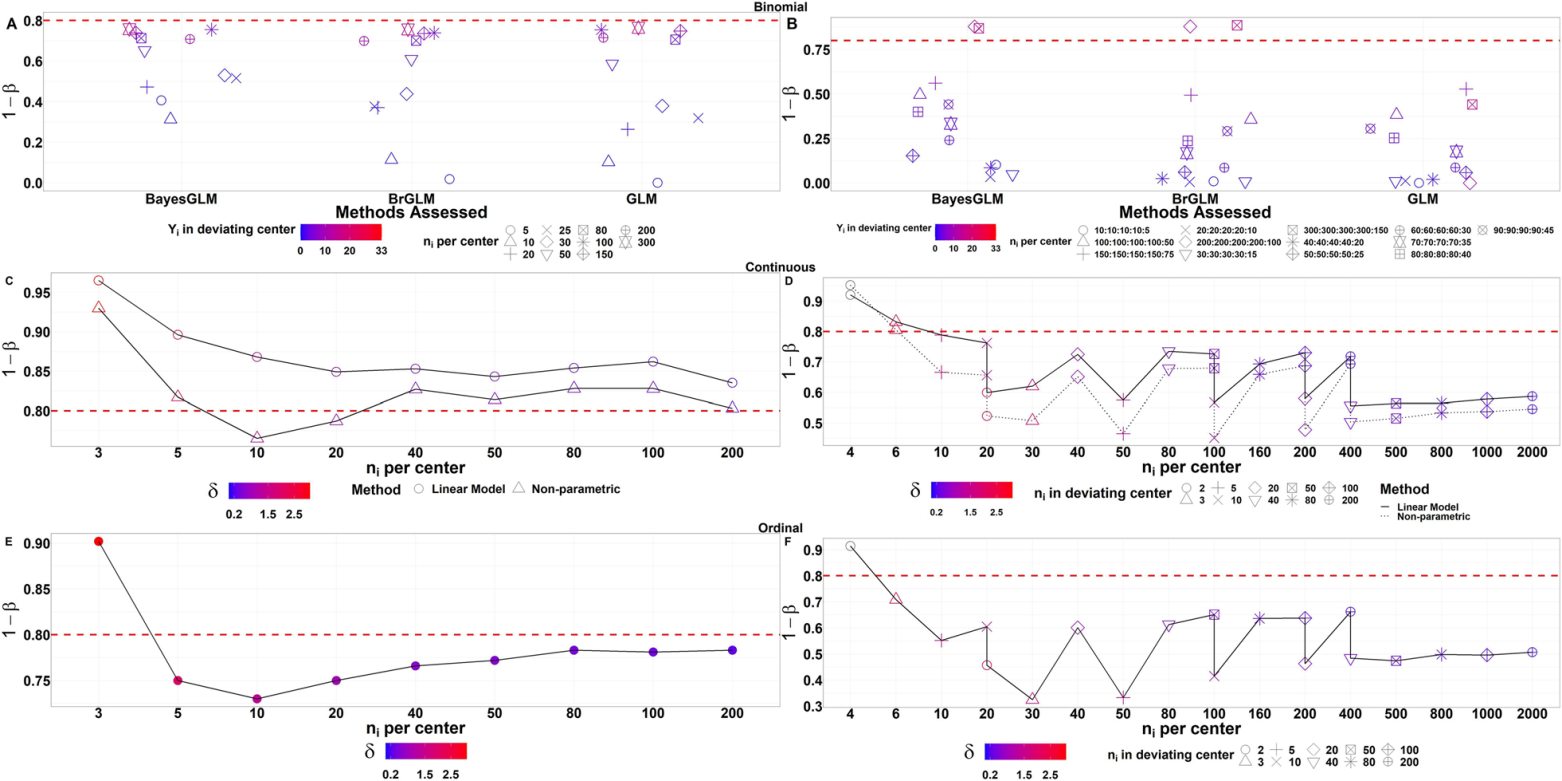
The Probability of Rejecting at Least One Null Hypothesis $(1-\beta )$ as a Function of Sample Size for Each Method Applied on Relevant Response Outcome for Balanced (Left Panel) and Unbalanced Designs (Right Panel). The group differences (*δ*) are chosen based on a two-sample test at a power level of 80%, which is shown as a horizontal line. BayesGLM Bayesian Generalized Linear Model, BrGLM Bias-reduced Generalized Linear Model, GLM Generalized Linear Model.

The power simulations for all methods are shown in Fig. 3. For a binomial outcome, Fig. 3a and b show the experimental setups used for balanced and unbalanced designs respectively. Methods show power increase as sample size per center *N* and success probability increase. Furthermore, BayesGLM is superior in power for small sample sizes relative to GLM and BrGLM, while controlling the type I error. Therefore, we recommend the use of BayesGLM for binomial outcomes that might contain rare events ${(Y}_{i}=0)$. For continuous outcome, similarly, to type I error simulations linear model was chosen as comparator to the non-parametric method. Figure 3c and d show power simulations of both methods for balanced and unbalanced scenarios respectively. Both methods achieve greater power for balanced designs than unbalanced ones. Additionally, the Non-parametric method has a trivial decrease in power compared to the linear model in all scenarios. Power rather decreases to ~ 50% for both methods in extreme settings of having small ${n}_{i}$ per center. For ordinal outcome, Fig. 3e and f show the power simulations of the non-parametric method for balanced and unbalanced experimental designs respectively. For balanced designs as the ${n}_{i}$ per center increases the power of the non-parametric method increases as well. Power decreases substantially for extreme settings of having small ${n}_{i}$ per center in which it reaches a maximum of 35%.

## Application to the GMSR Dataset

**Figure 4:**
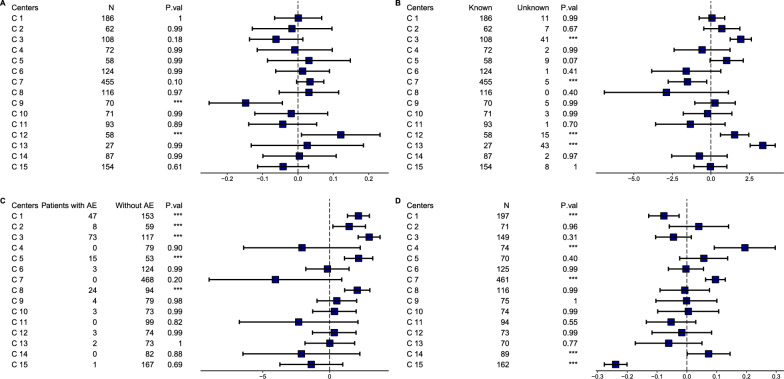
Simultaneous Confidence Intervals for Contrasts of Center Means with GM for GMSR Data Set. **A** Continuous variable age onset comparison for each center to GM using non-parametric method. **B** Fitting a BayesGLM for the binary variable of missingness of the age at onset followed by contrasts of center means towards GM. **C** Fitting a BayesGLM for AEs as binary variable followed by contrasts of center means and GM. **D** Ordinal variable of disease severity (EDSS) comparison for each center to the GM using the non-parametric method. BayesGLM Bayesian generalized linear model, AE Adverse events, GM Grand mean, ***significant *p* < 0.05.

We illustrate the proposed methods (except GLM and BrGLM as they show inferiority to BayesGLM) by an analysis of GMSR data. Methods were implemented on the corresponding variable type as appropriate. Figure 4 shows simultaneous confidence intervals of the deviations of the 15 centers from GM, for continuous, binary, and ordinal outcomes.

The non-parametric method was applied on the age at onset variable (Fig. 4a), C9 shows a cohort relatively smaller than GM of other centers, whereas C12 shows a cohort larger than the GM. In both cases, it is not a foremost observation for the GMSR data as it does not have a specific inclusion criterion for age onset of patients. However, it could be imperative for clinical trials as they do have a detailed inclusion criterion. A binary variable was derived presenting a missing input of the age at onset variable for each patient (Fig. 4b). C3 shows that it has 149 patients, however 41 of them do not have age onset information, although it’s not uncommon to have missing data for some variables, C3 shows a higher average than the GM. C12 and C13 have a similar pattern to C3 where both have a higher mean than the GM. While C7 has only five patients with missing information out of 460 patients, it shows a smaller mean for missing information than the GM of other centers. In other words, C7 signifies a superior documentation for age onset than other centers. Another binary variable presenting adverse events (per patient) reported per center is presented in Fig. 4c. C1, C2, C3 and C5 show higher proportions of adverse events reported than the GM of all centers. Looking in more detail into the AEs documented, C3 reports COVID-19 vaccination reactions as adverse events. This shows a clear example of how centers could perform differently from other centers. The results could indicate the need for stakeholders to approach under reporting centers, in other words it would point the centers that have a significantly smaller average than the GM. Finally Fig. 4d shows the contrasts of the centers’ EDSS measurements to the GM. It shows how center’s cohort disease severity for the specific center is different. C4, C7 and C14 show significantly higher EDSS measurements than the GM, while C1 and C15 show a smaller one. These results may alert stakeholders to further investigate the reasons for such differences. Particularly for C7, as it includes a cohort with higher disease severity than average and yet they report fewer adverse events.

## Discussion

In this paper we present methods and their implementation to detect center(s) that differ from GM of other centers for a specific variable. The utilization of these procedures serves the aim of CSM in performing data quality checks to improve data integrity. It also minimizes the costs of data monitoring and improves their quality. We were able to show how different statistical methods can be implemented to identify centers in multi-center trials or registry data that might need additional training or is a candidate for on-site monitoring visits. The approach allows the recognition of centers that are significantly deviating from the average. This would eventually enable the monitoring teams to point their attention to problematic sites.

The three methods investigated for binomial data never strongly exceed type I error. Nevertheless, BayesGLM is superior to GLM and BrGLM in detecting a deviating center when ${n}_{ij}<50$. The fact that all three methods tend to be too conservative for small sample sizes and rare events resembles similar problems found for other binomial methods: Due to the discreteness of binomial data, various methods are reported to be either over-conservative or liberal depending on the specific method and parameter configuration [36–38]. The non-parametric method has harsh violations of the type I error control; especially for ${n}_{ij}<10$, and ordinal data. In other words, the non-parametric method can be applied for clinical trials and registries where centers do not have a relatively small sample size, i.e., centers should have at least 10 patients to identify a true deviation. Our results show that the non-parametric method may result in an increased rate of falsely detecting deviating centers, when sample sizes are small. In some cases, an alternative would be to choose a suitable data transformation followed by application of parametric methods. However, in other cases, like contamination with outliers, bi- or multi-modal distributions, transformation may not settle the problems and non-parametric methods may still be the best choice. Further, it should be noted that the simulation studies in this paper are not suitable for fairly comparing non-parametric with parametric methods, because situations where non-parametric methods may outperform parametric approaches have not been involved.

Desmet et al. (2014, 2017) proposed alternative approaches to detect deviating centers, with differing assumptions [18, 19]. They assume that some variability between centers has to be expected and is not of concern, particularly if the number of centers is large. Consequently, they focus on detecting the deviations of a small proportion of contaminated centers from the distribution of the large majority of centers. They cover the important cases of continuous data under the additional assumption of normally distributed center means in a mixed effect model [18], and of binomial data with the assumption of beta distributed variability between centers [19]. Conversely, the models underlying the methods in this paper make no assumptions on the distribution of center means and are currently available for a wider range of model types and distributional assumptions for the data, including the non-parametric approach. However, this comes at the price of overfitting and possibly flagging more deviating centers than necessary in cases where variability between centers is allowed, particularly in trials involving a large number of centers. Further research is needed to investigate the approach practically for large multicenter clinical trials covering 20–100 centers with many being very low recruiters.

As Buyse et al. (2020) indicates, the power of a statistical approach lays in performing statistical tests on all variables. This would lead into many numbers of tests conducted and thus the need to combine their conclusions [39]. For this reason, a scoring system for an individual center could be further developed for the assessment of the individual data type with appropriate method. Parameters of the scoring system must be individually weighted by stakeholders. Although clinical trials and registries are similar in many aspects, a robust scoring system must be adaptable to consider their differences [40, 41]. For example, the inclusion criteria of patients differ between both systems. The deviations found in clinical trials are relatively smaller than in registry data as the latter usually have less strict inclusion criteria. Alternatively, it is possible to assess each center for how many variables it has been flagged for and treat it as a binomial measure to finally compare the actual number of how many variables are differing from other centers (see Supplementary Table 1). In other situations, expert knowledge in the CSM team may be used to assess what level of deviations is still acceptable for what variable. The proposed methods are then a statistical tool to assess which centers are within or outside such a range of acceptable deviations for a given variable. In such situations, a method that automatically processes all variables might not be desirable.

Several straightforward extensions of the approach are available. First, in some situations, it might be known that some centers differ from others. For example, centers located at well-known university hospitals might differ in frequencies of disease severity scores from centers at smaller, local hospitals. This again might lead to differences between distributions or summary statistics of several further variables. If it is desired to account for such expected differences between centers, the comparisons to GM can be stratified by the type of center. Alternatively, variables that are known to reflect such expected differences between centers can be included as covariates into (generalized) linear models, such that the comparisons between centers are performed while accounting for the effect of the covariates. Second, there are several variable types for which comparisons to GM can be performed but are not mentioned in detail in this paper. Ordinal data like disease severity scores can be analyzed by cumulative link models [42] with centers (and possibly further covariates) as explanatory variables, such that the tendency to show higher or lower scores can be compared between centers. Additional approaches for ordinal data such as ordered categorical regression and multinomial models for nominal data are available. Time-to-event data or survival times are abundant in clinical trials, and multiple comparisons can be performed for such data, because the cox model as well as Weibull models for survival time are special cases of the framework implemented in the multcomp package [21]. Moreover, skewed continuous data can be modelled in generalized linear models assuming exponential, gamma, or inverse Gaussian distribution. Several types of heteroscedasticity can be modelled by generalized least square models. Again, for these model types, comparisons to Grand mean can be performed using the multcomp package. Further research is needed to assess the performance of these extensions for limited sample sizes for the investigated approach.

Currently methods are scattered between different packages in R. We provide an easy to use and interactive graphical user interface for the two methods BayesGLM & Nparcomp as two separate shinyapps, https://central-statistical-monitoring.shinyapps.io/BayesGLM-GM/ and https://central-statistical-monitoring.shinyapps.io/Nparcomp-GM/. Users can upload their datasets to compute comparisons to GM, and graphically represent simultaneous confidence intervals for contrasts of center means with GM. We plan to introduce a universal form of the methods demonstrated in a standard R package to tackle different data types easing their implementation and drawing respective decision charts for the benefit of CSM. Central statistical monitoring serves the core purpose of monitoring goals. It facilitates the detection of deviating centers that are not likely due to chance. This would eventually support monitoring teams to initiate an onsite visit and target their activities.

## Supplementary

The Syntax of all simulations and respective datasets as well as for the shinyapps are available on Github under https://github.com/firasfneish/CSM.

### Supplementary Information

Below is the link to the electronic supplementary material.

Supplementary file1 (PDF 1399 KB)

Supplementary file2 (DOCX 21 KB)
