# Supplementary material for: Application of Statistical Methods for Central Statistical Monitoring and Implementations on the German Multiple Sclerosis Registry
Source: Ther Innov Regul Sci. 2023 Jul 14;57(6):1217–28. doi: 10.1007/s43441-023-00550-0 (PMC10579126; doi:10.1007/s43441-023-00550-0)
Supplement: Supplementary file 1 — Supplementary file1 (PDF 1399 KB) [file 43441_2023_550_MOESM1_ESM.pdf]

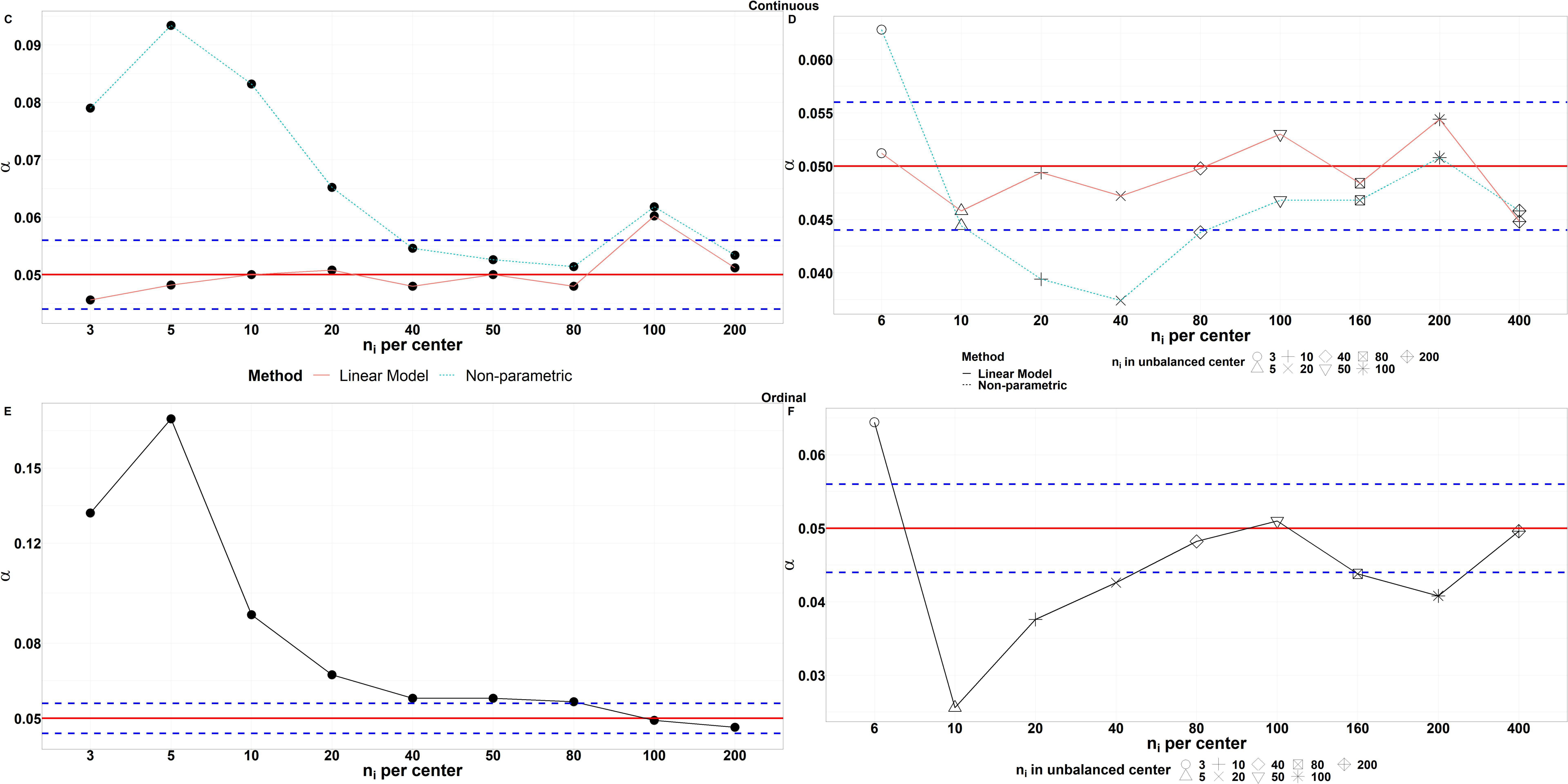
Continuous
C
α
0.09
0.08
0.07
0.06
0.05
3 5 10 20 40 50 80 100 200
n_i per center
Method Linear Model Non-parametric
D
α
0.060
0.055
0.050
0.045
0.040
6 10 20 40 80 100 160 200 400
n_i per center
Method
Linear Model
Non-parametric
n_i in unbalanced center 3 5 10 20 40 50 80 100 200
Ordinal
E
α
0.15
0.12
0.08
0.05
3 5 10 20 40 50 80 100 200
n_i per center
F
α
0.06
0.05
0.04
0.03
6 10 20 40 80 100 160 200 400
n_i per center
n_i in unbalanced center 3 5 10 20 40 50 80 100 200

Supplementary figure 1: The probability of falsely rejecting the null hypothesis for at least one center as a function of sample size for each method applied on relevant response outcome for balanced (left panel) and unbalanced designs (right panel). The nominal type I error rate ($\alpha = 0.05$) is shown as a horizontal line. Dotted blue lines indicate error margins for simulations with 5000 runs. Simulated type-I-errors falling outside [0.044; 0.056] indicate a significant deviation from the prespecified level ($\alpha = 0.05$).
