# Supplementary material for: Application of Statistical Methods for Central Statistical Monitoring and Implementations on the German Multiple Sclerosis Registry
Source: Ther Innov Regul Sci. 2023 Jul 14;57(6):1217–28. doi: 10.1007/s43441-023-00550-0 (PMC10579126; doi:10.1007/s43441-023-00550-0)
Supplement: Supplementary file 2 — Supplementary file2 (DOCX 21 KB) [file 43441_2023_550_MOESM2_ESM.docx]

**Supplementary Table S1.** Summarized table for flagged variables in each center

|  | Age onset | Age onset missing | Adverse events | Disease severity | Total variables flagged for |
| --- | --- | --- | --- | --- | --- |
| C1 | 0 | 0 | 1 | 1 | 2 |
| C2 | 0 | 0 | 1 | 0 | 1 |
| C3 | 0 | 1 | 1 | 0 | 2 |
| C4 | 0 | 0 | 0 | 1 | 1 |
| C5 | 0 | 0 | 1 | 0 | 1 |
| C6 | 0 | 0 | 0 | 0 | 0 |
| C7 | 0 | 1 | 0 | 1 | 2 |
| C8 | 0 | 0 | 1 | 0 | 1 |
| C9 | 1 | 0 | 0 | 0 | 1 |
| C10 | 0 | 0 | 0 | 0 | 0 |
| C11 | 0 | 0 | 0 | 0 | 0 |
| C12 | 1 | 1 | 0 | 0 | 2 |
| C13 | 0 | 1 | 0 | 0 | 1 |
| C14 | 0 | 0 | 0 | 1 | 1 |
| C15 | 0 | 0 | 0 | 1 | 1 |
